# E-prescribing and medication safety in community settings: A rapid scoping review

**DOI:** 10.1016/j.rcsop.2023.100365

**Published:** 2023-11-07

**Authors:** Christine E. Cassidy, Leah Boulos, Erin McConnell, Brittany Barber, Alannah Delahunty-Pike, Andrea Bishop, Nawal Fatima, Amanda Higgins, Megan Churchill, Allison Lively, Shannon P. MacPhee, Ruth Martin Misener, Rowan Sarty, Robert Wells, Janet A. Curran

**Affiliations:** a Dalhousie University, Halifax, Nova Scotia, Canada; b IWK Health, Halifax, Nova Scotia, Canada; c Maritime SPOR SUPPORT Unit, Nova Scotia Health, Halifax, Nova Scotia, Canada; d Nova Scotia College of Pharmacists, Halifax, Nova Scotia, Canada; e Nova Scotia Health, Halifax, Nova Scotia, Canada; f Nova Scotia Department of Health and Wellness, Halifax, Nova Scotia, Canada; g Patient Partner, Halifax, Nova Scotia, Canada

**Keywords:** Scoping review, Medication errors, Medication safety, Pharmacy

## Abstract

**Background:**

Medication prescribing is essential for the treatment, curing, maintenance, and/or prevention of an illness and disease, however, medication errors remain common. Common errors including prescribing and administration, pose significant risk to patients. Electronic prescribing (e-prescribing) is one intervention used to enhance the safety and quality of prescribing by decreasing medication errors and reducing harm. *E*-prescribing in community-based settings has not been extensively examined.

**Objective:**

To map and characterize the current evidence on e-prescribing and medication safety in community pharmacy settings.

**Methods:**

We conducted a rapid scoping review of quantitative, qualitative, and mixed methods studies reporting on e-prescribing and medication safety. MEDLINE All (OVID), Embase (Elsevier), CINAHL Full Text (EBSCOHost), and Scopus (Elsevier) databases were searched December 2022 using keywords and MeSH terms related to e-prescribing, medication safety, efficiency, and uptake. Articles were imported to Covidence and screened by two reviewers. Data were extracted by a single reviewer and verified by a second reviewer using a standardized data extraction form. Findings are reported in accordance with JBI Manual for Evidence Synthesis following thematic analysis to narratively describe results.

**Results:**

Thirty-five studies were included in this review. Most studies were quantitative (*n* = 22), non-experimental study designs (*n* = 16) and were conducted in the United States (*n* = 18). Half of included studies reported physicians as the prescriber (*n* = 18), while the remaining reported a mix of nurse practitioners, pharmacists, and physician assistants (*n* = 6). Studies reported on types of errors, including prescription errors (*n* = 20), medication safety errors (*n* = 9), dispensing errors (*n* = 2), and administration errors (*n* = 1). Few studies examined patient health outcomes, such as adverse drug events (*n* = 5).

**Conclusions:**

Findings indicate that most research is descriptive in nature and focused primarily on rates of prescription errors. Further research, such as experimental, implementation, and evaluation mixed-methods research, is needed to investigate the effects of e-prescribing on reducing error rates and improving patient and health system outcomes.

## 1 Introduction

Prescribing is an essential tool used by clinicians (e.g., nurse practitioners, pharmacists, physicians, physician assistants) to continue the use of/order a medication or intervention for a patient and to provide directions in the treatment, curing, maintenance, and/or prevention of an illness or disease.^1^, 2., 3. When prescribing, clinicians must use professional judgment and follow professional standards to ensure patient and medication safety.^1^^,^^3^ It is essential that prescriptions clearly define the right patient, right medication, right dose, right duration, and right route; otherwise, potential harm can be caused to patients, and if serious, can be fatal.^4^^,^^5^ Traditionally, prescriptions were written by hand, or faxed, therefore, illegible prescriptions increased the risk of medication errors.^6^

In Canada, approximately 7.5% of all hospital admissions in an intensive care unit experience adverse events as a result of medication errors,^7^ and approximately 1 in 30 patients are exposed to medication harm globally.^8^ Despite the prevalence of medication-related harm, most errors could be prevented.^8^ A systematic review and meta-analysis on preventable medication harm in hospital settings shows a majority of medication errors occur at the prescribing (58%, 42 to 73%, *n* *=* 9, *I*^*2*^ *=* 94%) and monitoring (47%, 21 to 73%, *n* *=* 8, *I*^*2*^ = 99%) stages of medication use.^8^ Common medication errors including prescribing and administration, pose significant risk to vulnerable and critically ill patients, particularly for pediatric^9^ and elderly^10^ patients that have the highest rates of preventable medication harm.^8^^,^^11^ In 2018, researchers analyzed medication errors that reached patients through community-based pharmacies in Nova Scotia, Canada.^12^ The most common medication errors found included incorrect dose or frequency (27.4%; *n* = 254), incorrect strength or concentration (20.2%; *n* = 187), and incorrect drug (19.9%; *n* = 185).^12^ Medication errors in both hospital and community-based settings have a direct impact on the health system such as, increased rehospitalization rates, prolonged length of stay, and increased cost of hospitalization.^13^

Electronic prescribing (e-prescribing) is one intervention strategy used to enhance the safety and quality of the prescribing process by decreasing medication errors and reducing harm.^14^ *E*-prescribing is the electronic transmission of a prescription between an authorized prescriber and pharmacist using an electronic medical record or pharmacy management software.^15^ The potential benefits of e-prescribing include simplifying and improving the process of submitting a prescription to a pharmacy to facilitate efficient dispensing and refills.^16^^,^^17^ *E*-prescribing can also facilitate important information sharing between providers and across multiple pharmacies.^16^ For example, e-prescribing has shown to decrease risk of medication errors (99% relative risk reduction) and adverse drug events (98% relative risk reduction) in hospital settings and outpatient settings.^18^^,^^19^

Use of e-prescribing has increased substantially over the past fifteen years.^20^^,^^21^ In hospital settings, the uptake of e-prescribing has increased ten-fold between 2008 and 2014 from 7 to 70%.^21^ However, in community-based settings such as primary care clinics, uptake of e-prescribing is limited.^22^ Barriers to implementing e-prescribing within community-based clinical settings include cost, lack of provider support, patient privacy, system errors, and legal issues.^14^^,^^23^ Uptake of e-prescribing in community-based clinical settings is more common for young early career physicians, pediatricians, and large clinical practices with more than 9000 patients.^22^ As more patients have access to virtual care,^16^ e-prescribing is an important tool that allows patients to receive their prescriptions at a pharmacy of their choosing close to home.^21^

Uptake of e-prescribing in community-based settings is important for improving medication safety and efficiency, improving patient satisfaction, preventing medication error harms, and reducing health system costs from hospital admission/readmission rates.^14^^,^^19^^,^^24^ Despite extensive research on e-prescribing and medication safety in hospital settings; e-prescribing and medication safety in community-based settings has not been systematically examined.

### 1.1 Review objectives

The primary objective of this review was to map and characterize the current evidence on e-prescribing and medication safety in community-based settings. The secondary objective was to identify outcomes related to prescriber uptake of e-prescribing, efficiencies, and process improvements.

## 2 Methods

This rapid scoping review was guided by the Joanna Briggs Institute (JBI) Manual for Evidence Synthesis, with modifications made due to the rapid timeline based on Tricco (2017) guide to rapid reviews.^25^^,^^26^

### 2.1 Search strategy

A health information specialist (LB) designed the initial search in MEDLINE All (Ovid) using keywords and MeSH terms relating to e-prescribing, medication safety, efficiency, and uptake. The search terms were developed after conducting preliminary scoping searches in November 2022, consulting with team members and subject matter experts, and mining appropriate terminology from previously identified literature on the topic. No search hedges, study design filters, language or date limits were applied to the search. A second information specialist (MR) reviewed the MEDLINE search strategy using the Peer Review of Electronic Search Strategies (PRESS) guidelines prior to translation to other databases.^27^

### 2.2 Information sources

After peer review, the MEDLINE search strategy was translated to Embase (Elsevier), CINAHL Full Text (EBSCOHost), and Scopus (Elsevier). The search strategy for all databases is included in Supplementary Material File 1. Given the timeline for this rapid review, a separate grey literature search was not conducted, but some grey literature and preprints were captured by the database searches. All searches were executed on December 15, 2022.

### 2.3 Inclusion criteria

#### 2.3.1 Participants

This review considered all literature on physicians, nurse practitioners, or pharmacists as primary users of the e-prescribing system. This review also considered all patient groups.

#### 2.3.2 Concept

The primary concept of interest for this review was medication safety in the context of e-prescribing. *E*-prescribing was described as a tool that enables prescribers to transmit prescriptions electronically and communicate with pharmacists. Articles that only reported on electronic transmission to fax were excluded. Medication safety included communication/transcribing errors, adverse drug events, or inappropriate prescribing. A secondary concept of interest for this review was health system, prescriber, or patient factors associated with efficiency in e-prescribing or uptake of e-prescribing. The primary concept was required for a study's inclusion in the review, while the secondary concept was optional for inclusion.

#### 2.3.3 Context

This review included articles where e-prescribing took place between clinician prescribers and community pharmacists. This may have included e-prescribing in primary or ambulatory care clinics or during care transitions from hospital to home. Studies were excluded from this review if the pharmacy was in a hospital or institutional setting.

### 2.4 Types of sources

This review considered experimental, non-experimental, quasi-experimental, mixed methods, or qualitative studies for inclusion. Descriptive studies with no evaluation component, commentaries, discursive papers, and other publications that did not present original data were excluded from this review. Evidence syntheses were excluded from this review; however, potentially relevant evidence syntheses were tagged and their included studies were screened for inclusion in this review. Non-English records and conference abstracts were excluded during screening.

### 2.5 Study selection

Records retrieved by the database searches were exported to RIS format. In Scopus, MEDLINE and Embase records were removed prior to export using the search string *AND NOT ((INDEX(medline) OR (INDEX(embase))).* All remaining citations from each database were imported into Covidence for removal of duplicates and screening. Following scoping review guidelines,^25^^,^^26^ citations were screened independently by two reviewers at both the title/abstract and full text levels. Any conflicts that arose were resolved by a third reviewer or by consensus.

### 2.6 Data extraction

Data extraction forms were created in Microsoft Excel by the research team in consultation with a panel of content experts (Supplementary Material File 2) and piloted with a sample of included studies by two independent reviewers. Data extracted included date of publication, setting and time covered, study design, description of e-prescribing system, key outcome(s), and summary of key findings in relation to the outcome(s). Data were extracted by a single reviewer and verified independently by a second reviewer. Primary outcome definitions are described in Table 1.

**Table 1:** Primary outcome definitions.

**Primary Outcomes**	**Definition**
**Prescription errors**	Errors associated with the prescribing process and/or the prescription^28^
**Medication safety errors**	Errors with the potential for harm^29^
**Dispensing errors**	Errors associated how the medication is dispensed at the pharmacy^30^
**Administrative errors**	Errors associated with patient insurance and/or affordability^31^
**Patient safety**	Outcomes with implications for broader patient safety^32^
**Adverse drug events**	Harm caused by medication or lack of intended medication^33^

### 2.7 Data analysis

The Canadian Medication Incident Reporting and Prevention System (CMIRPS) classification system was used to better understand the type of quality-related event.^34^ Quantitative data were summarized in tabular format and qualitative data were summarized narratively.

### 2.8 Knowledge user and patient/public involvement

Members of the review team met biweekly with a panel of content experts including pharmacists, clinicians, and a Patient/Public Partner to ensure alignment with their experiences and to learn from their expertise on e-prescribing. Content experts contributed to designing the review questions, inclusion/exclusion criteria, and data extraction form, and supported interpretation of the study findings.

## 3 Results

### 3.1 Characteristics of included studies

**Fig. 1:**
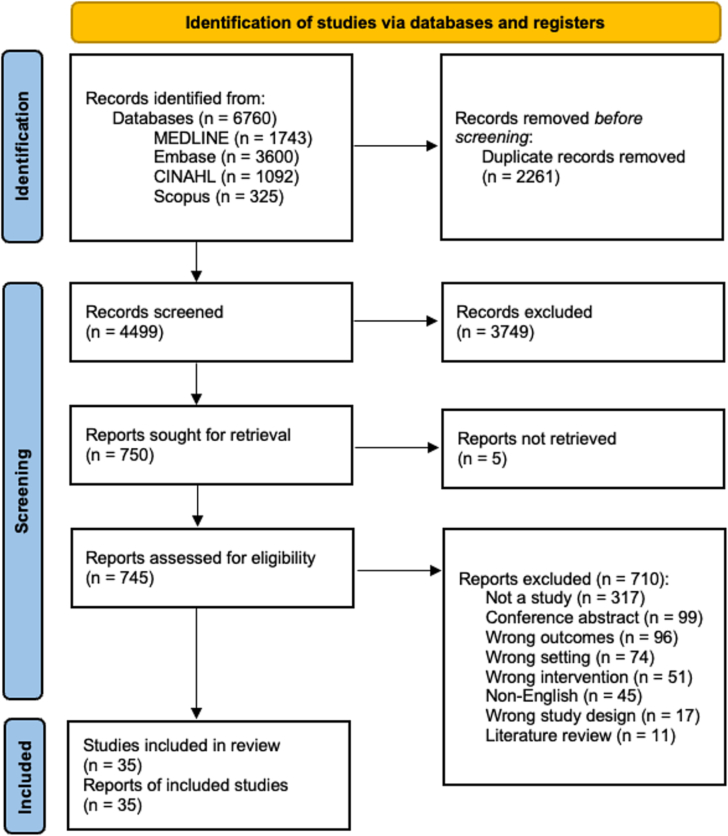
PRISMA flow diagram.

Thirty-five studies were included in this review. There were 6760 studies identified in the database search; and 2261 duplicates removed prior to screening. A total of 4499 studies were screened at the title/abstract level and 750 studies screened at the full-text level. The final number of included and excluded articles, with reasons for exclusion, is reported in a PRISMA diagram (Fig. 1). An overview of included study characteristics and a summary of key findings is presented (see Supplementary Material File 3).

Thirty-five included studies (quantitative: *n* = 22; qualitative: *n* = 8; mixed methods: *n* = 5) were published between 2007 and 2022, with 10 studies published since 2018 (29%). Of the 21 quantitative studies, 16 used non-experimental designs and 3 used quasi-experimental designs. Only 2 studies used experimental designs to evaluate the effect of e-prescribing on prescription and/or dispensing errors compared to hand-written or printed prescriptions.

The United States was the most advanced country (*n* = 18) in reporting e-prescribing and medication safety, representing 51% of all included studies.^31^^,^^35^, ^36^, ^37^, ^38^, ^39^, ^40^, ^41^, ^42^, 43., 44, 45, 46, 47, 48, 49, 50, 51 Other countries included Finland (*n* = 3),^52^, ^53^, ^54^ Australia (*n* = 2),^55^^,^^56^ Sweden (*n* = 2),^57^^,^^58^ Norway (*n* = 2),^59^^,^^60^ United Kingdom (*n* = 1),^61^ Belgium (*n* = 1),^62^ Malaysia (*n* = 1),^63^ Jordan (*n* = 1),^64^ Saudi Arabia (*n* = 1),^65^ Lebanon (*n* = 1),^66^ and Canada (*n* = 1).^67^ One study (*n* = 1) did not report location.^68^ Half of the included studies (*n* = 18, 51%) reported physicians as the prescriber, whereas the remaining studies reported a mix of prescribers including nurse practitioners, pharmacists, and physician assistants. A small number of studies described e-prescribing as integrated within electronic health record systems (*n* = 10). *E*-prescribing systems with a pull model were seen in 6 studies (*n* = 6),^52^, ^53^, ^54^^,^^57^^,^^58^^,^^60^ where prescriptions are sent electronically to a centralized e-prescription database that can be accessed by any pharmacist.^69^ Moreover, seven studies (*n* = 7) described a push system, where e-prescriptions are sent to a specific pharmacy.^35^^,^^36^^,^^47^^,^^50^^,^^56^^,^^61^^,^^62^ However, the majority of studies did not report the type of push/pull system (*n* = 22).^31^^,^^37^, ^38^, ^39^, ^40^, ^41^, ^42^, 43., 44, 45, 46^,^^48^^,^^49^^,^^51^^,^^55^^,^^59^^,^63., 64, 65, 66, 67, 68

### 3.2 Medication safety

**Fig. 2:**
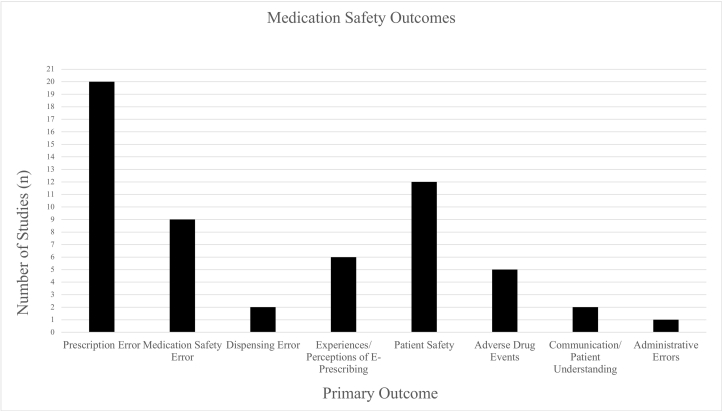
Medication safety outcomes.

Almost all studies reported on types of errors, including prescription errors (*n* = 20),^31^^,^^36^, ^37^, ^38^, ^39^, ^40^^,^^42^^,^^44^^,^^46^^,^^49^^,^^51^^,^^53^^,^^54^^,^^56^^,^^61^^,^^62^^,^^64^, ^65^, ^66^^,^^68^ medication safety errors (*n* = 9) (40,48,53,54,58,59,63,67), dispensing errors (*n* = 2),^31^^,^^64^ and administration errors (*n* = 1)^31^ (Fig. 2). The majority of quantitative and mixed methods studies (*n* = 15) used frequency counts as their primary outcome measure to assess error rates. The most common types were incorrect dose and frequency (*n* = 5), followed by incorrect strength/concentration (*n* = 4), and incorrect route of administration (*n* = 3).

Fewer studies examined e-prescribing and patient health outcomes, including adverse drug events (*n* = 5).^35^^,^^36^^,^^38^^,^^40^^,^^43^ For example, Nanji et al. (2011) examined outpatient computerized prescribing systems across 3 American states to identify incidence of medical errors, potential adverse drug events, and rate of prescribing errors by error type and prescribing system.^40^ In a sample of 3850 prescriptions, 452 (12%) contained a total of 466 errors. Researchers classified 163 (35%) of these errors as potential adverse drug events, indicating that 4% of prescriptions contained potential adverse drug events. Of the potential adverse drug events, 95 (58%) were significant, 68 (42%) were serious, and none were life-threatening.^40^

Other reported medication safety outcomes include overall patient safety (*n* = 12)^35^^,^^41^^,^^45^, ^46^, ^47^^,^^50^, ^51^, ^52^^,^^55^^,^^57^^,^^60^^,^^66^ without a description of patient health outcomes; communication/patient understanding (*n* = 2)^41^^,^^49^; and experiences/perceptions of e-prescribing (*n* = 6).^45^, ^46^, ^47^^,^^50^^,^^53^^,^^60^ Greater details of studies reporting medication safety outcomes can be found in Supplementary Material File 4.

### 3.3 Efficiency, process improvement, and uptake outcomes

**Fig. 3:**
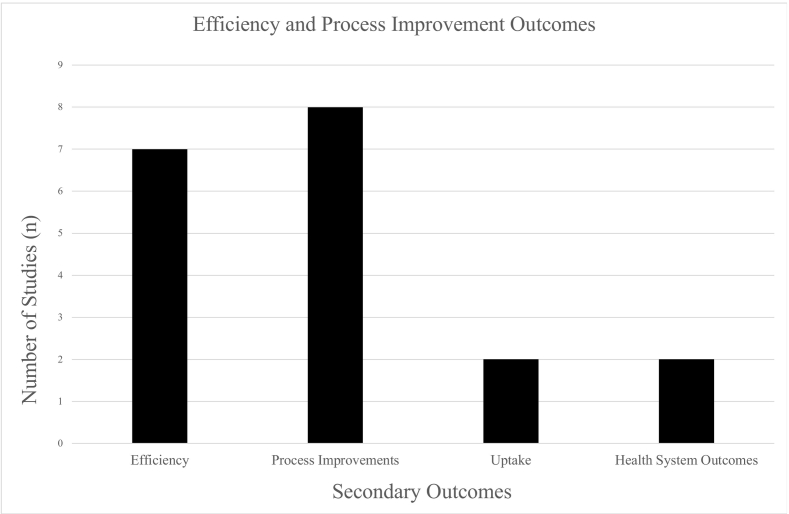
Efficiency and process improvement outcomes (*n* = 15/35).

Fifteen studies (*n* = 15) measured outcomes related to efficiency, process improvements, uptake, and health system outcomes (Fig. 3).

The most common secondary outcome reported was efficiency (*n* = 7)^31^^,^^45^^,^^46^^,^^49^^,^^51^^,^^56^^,^^57^ Four of these studies reported efficiencies gained by e-prescribing,^45^^,^^46^^,^^56^^,^^57^ four studies reported inefficiencies with e-prescribing.^31^^,^^46^^,^^49^^,^^51^

Eight studies (*n* = 8) reported process improvements with e-prescribing,^46^^,^^52^^,^^53^^,^^57^^,^^58^^,^^63^^,^^67^^,^^68^ such as ease of use (*n* = 3),^52^^,^^63^^,^^68^ communication (*n* = 2),^57^^,^^63^ work efficacy and productivity (*n* = 1).^58^^,^^67^ Three qualitative studies explored e-prescribing system improvements and identified the need to improve technological infrastructure to optimize e-prescribing.^52^^,^^53^^,^^57^

Two studies (*n* = 2) examined e-prescribing uptake from the provider and patient perspective.^41^^,^^56^ Elliott (2016) found limited uptake of e-prescribing among physicians, whereas increased uptake was found among nurses and pharmacists.^56^ Further, Bergeron (2013) examined rates of prescription abandonment among patients and found that many patients were not picking up e-prescriptions from the pharmacy due to medication cost/lack of insurance coverage, choosing over-the-counter medication instead, or wanting to see if their condition improved.^41^

Two studies (*n* = 2) examined e-prescribing and health system outcomes, including improved cost effectiveness (*n* = 2)^35^^,^^57^ and improved healthcare utilization (*n* = 1) (i.e., fewer hospitalizations and fewer emergency department and office visits).^35^ More information on secondary outcomes can be found in Supplementary Material File 5.

## 4 Discussion

This review identified 35 studies reporting on e-prescribing and medication safety in community-based settings. Most of the included studies used non-experimental, quantitative research designs with a focus on describing rates of prescription errors. Few studies measured patient and health system outcomes directly. When it comes to e-prescribing, physicians are the most common e-prescriber; however, 17 of the included studies reported on a mix of e-prescribers including nurse practitioners, physician assistants, and pharmacists. Moreover, close to half of the included studies examined efficiency and uptake of e-prescribing and reported a mix of efficiencies and inefficiencies with e-prescribing. Some studies examined the impact of e-prescribing on medication safety, indicating positive trends in reducing prescription and dispensing errors and improvements in patient safety and communication.

This rapid scoping review highlights the need for more experimental research on the direct impacts of e-prescribing on medication safety. While the majority of studies included in this scoping review were quantitative designs (*n* = 22), most were non-experimental (*n* = 16) or quasi-experimental designs (*n* = 3); very few studies (*n* = 2) used experimental designs. Given the limitations of observational study designs on evaluating the impact of e-prescribing, future research would benefit from using experimental study design to investigate the effect of e-prescribing on the medication safety outcomes identified in this review.

Efforts are also needed to examine implications of e-prescribing on patient health outcomes beyond prescription errors. Most studies included in this review examined the rate of prescription errors rather than the impact of prescription errors on patient and health system outcomes. Studies reporting on e-prescribing in ambulatory care settings show improved patient safety, improved legibility, reduced time between prescribing and dispensing of medications, and fewer medication errors and adverse drug events.^14^^,^^19^ Despite these improvements, adverse outcomes from e-prescription errors in hospital settings continue to have direct negative impacts on patients and the health system.^19^ Adverse outcomes include mortality, increased frequency and length of hospitalizations, failure of treatment, and side effects such as increased pain, nausea and vomiting, and risk of infection.^19^ Further research is needed to understand contexts influencing adverse outcomes from e-prescribing in comparison to traditional paper prescribing methods and potential strategies to mitigate these negative impacts.

Pull (*n* = 6) and push (*n* = 7) models were 2 different types of e-prescribing models identified in this review. Although half of included studies described either pull or push models, details were limited on the impacts of each model on medication safety, including benefits/challenges to each type of model. Improved reporting of e-prescribing interventions is needed, including descriptions of the type of pull or push e-prescription model, as well as type of e-prescribing technology used, to inform future design, implementation, scale, and spread of e-prescribing interventions.

There was a strong focus on physicians as prescribers in the included studies; however, there is a need for examination of e-prescribing practices with other prescribers. For example, prescribing has been added to the scope of practice of clinicians including nurse practitioners, pharmacists, and registered nurses.^70^, ^71^, ^72^, ^73^ While patients and clinicians report positive outcomes from their expanded scope of practice,^71^^,^^72^ it is unclear how e-prescribing influences patient care, communication between multiple interprofessional clinicians, and integration across health services with different electronic health record systems.

Findings from this rapid scoping review provide important insight on the range of medication safety outcomes to measure, technological barriers to address, and system process considerations. While there are multiple available e-prescribing systems, those identified in this review include Electronic Prescription Service,^61^ Prescription Centre,^52^, ^53^, ^54^ National Online Prescription Repository,^57^ Integrated electronic prescribing system,^58^ PocketScript,^35^ SureScripts,^39^^,^^50^ and Prescription Mediator.^60^ Although studies described the characteristics of these e-prescribing systems, there was a lack of description of how to implement and sustain these systems in practice. It is anticipated that each system and local implementation context may have their own unique barriers and facilitators to implementation and sustainability. Gagnon and colleagues (2013) conducted a systematic review on the barriers and facilitators to implementing e-prescribing in primary care settings.^74^ Barriers to implementing e-prescribing identified software/hardware problems with the system, irrelevant alerts related to medications, and patient medical information not updating as needed (e.g. prescribing history).^23^^,^^74^ Similarly, research has found that technology has been a barrier to e-prescribing implementation among pharmacists (in both community and hospital settings), due to loss of working hours to deal with technological problems.^23^^,^^58^^,^^75^ Facilitators associated with implementing e-prescribing include prescribers seeing the value to e-prescribing, technology being familiar, patients' attitudes, and efficiency.^74^ Improved reporting of e-prescribing interventions in the published literature is needed (including the type of push or pull e-prescription model, type of e-prescribing technology), to inform the future design, implementation, scale, and spread of e-prescribing interventions. Building on this work, additional research designs (e.g., qualitative, mixed methods) could expand our understanding of how and why e-prescribing impacts medication safety, patient outcomes, and prescriber behaviours. We recommend embedding implementation science methods into future e-prescribing research to enhance the acceptance, uptake, and sustainability of e-prescribing interventions in community-based pharmacy settings.

### 4.1 Limitations

Although our approach to searching and screening was comprehensive and systematic by following the discussed scoping review guidelines, we only included English-language studies during screening due to the short timeline and limited non-English language proficiencies of our research team. This may have resulted in the omission of relevant literature published in other languages. Grey literature searching was not undertaken due to time constraints and the low likelihood of retrieving additional relevant studies.

## 5 Conclusion

This rapid scoping review mapped and characterized the current evidence on e-prescribing and medication safety in community-based settings, and prescriber uptake and efficiency outcomes related to e-prescribing. Findings indicate that most of the reviewed research is non-experimental in nature and focused primarily on rates of prescribing errors. Further experimental research is needed to investigate the effects of e-prescribing on reducing error rates and improving patient and health system outcomes. This rapid scoping review provides important insight on the range of medication safety outcomes, technological barriers, and system process considerations. Future research would benefit from including detailed descriptions on the processes of implementing e-prescribing to enhance the acceptance, uptake, and sustainability of e-prescribing interventions in community-based pharmacy settings.

## Authors' contributions

CEC, LB, ADP, AB, AL, SAM, RMM, RS, RW, JAC contributed to study conceptional, methodology, and formal analysis. CEC, LB, EM, ADP, NF, AH, MC contributed to data curation and formal analysis. CEC, LB, EM, ADP, JAC wrote the original draft. All authors critically reviewed and edited the manuscript and approved the final version.

## Funding acknowledgement(s)

This independent study was funded by Canada Health Infoway Inc. and brokered by the SPOR Evidence Alliance (SPOR EA), which is supported by the 10.13039/501100000024Canadian Institutes of Health Research (CIHR) under the Strategy for Patient-Oriented Research (SPOR) initiative.

## General disclaimer

This report was prepared by the co-authors on behalf of the SPOR Evidence Alliance for Canada Health Infoway Inc. It was developed through the analysis, interpretation and synthesis of scientific research and/or health technology assessments published in peer-reviewed journals. It also incorporates selected information provided by experts and patient partners with lived experience on the subject matter. This document may not fully reflect all the scientific evidence available at the time this report was prepared. Other relevant scientific findings may have been reported since completion of this synthesis report.

SPOR Evidence Alliance and the project team make no warranty, express or implied, nor assume any legal liability or responsibility for the accuracy, completeness, or usefulness of any information, data, product, or process disclosed in this report. Conclusions drawn from, or actions undertaken on the basis of, information included in this report are the sole responsibility of the user. The opinions, results, and conclusion reported in this paper are those of the authors. No endorsement by Canada Health Infoway Inc. is intended or should be inferred.

## Declaration of Competing Interest

The authors declare that they have no conflict of interests.

## Appendix A Supplementary data

Supplementary material Image 1
