# Supplementary material for: E-prescribing and medication safety in community settings: A rapid scoping review
Source: Explor Res Clin Soc Pharm. 2023 Nov 7;12:100365. doi: 10.1016/j.rcsop.2023.100365 (PMC10679534; doi:10.1016/j.rcsop.2023.100365)
Supplement: Supplementary file 1 — Supplementary material [file mmc1.docx]

**Supplementary Material File 1: Search Strategy**

All searches were executed on December 15, 2022.

**MEDLINE All (Ovid)**

| 1 | Electronic Prescribing/ | 1163 |
| --- | --- | --- |
| 2 | (eprescrib* or eprescription*).ti,ab,kf. | 107 |
| 3 | (e prescrib* or e prescription*).ti,ab,kf. | 572 |
| 4 | (epharmacy or epharmacies).ti,ab,kf. | 0 |
| 5 | (e pharmacy or e pharmacies).ti,ab,kf. | 28 |
| 6 | ((computeri?ed or digital* or electronic* or internet or online or virtual*) adj2 (prescrib* or prescription*)).ti,ab,kf. | 2349 |
| 7 | ((computeri?ed or digital* or electronic* or internet or online or virtual*) adj2 (pharmacy or pharmacies)).ti,ab,kf. | 875 |
| 8 | (electronic* adj2 (transmit* or transmission or send* or sent) adj2 (prescrib* or prescription*)).ti,ab,kf. | 57 |
| 9 | ((pharmac* or prescrib* or prescription*) adj3 software*).ti,ab,kf. | 259 |
| 10 | or/1-9 | 4232 |
| 11 | Inappropriate Prescribing/ | 4459 |
| 12 | Medical Errors/ | 17645 |
| 13 | exp Medication Errors/ | 19778 |
| 14 | Patient Harm/ | 210 |
| 15 | Patient Safety/ | 25033 |
| 16 | Safety/ | 41841 |
| 17 | adverse event*.ti,ab,kf. | 211272 |
| 18 | ((clinician* or doctor* or drug* or medical or medication* or medicin* or pharmac* or physician* or practitioner* or prescrib* or prescription* or provider* or treatment*) adj3 (accident* or error* or inadvertent* or mistake* or uninten*)).ti,ab,kf. | 25318 |
| 19 | (decei* or fake or fraud* or misrepresent* or scam* or sham).ti,ab,kf. | 112194 |
| 20 | ((drug* or medical or medication* or medicin* or patient* or prescrib* or prescription*) adj3 (safe or safeguard* or safely or safeties or safety)).ti,ab,kf. | 108242 |
| 21 | ((drug* or medication* or medicin* or treatment*) adj3 (misuse* or overuse)).ti,ab,kf. | 6560 |
| 22 | (harm or harmed or harmful* or harms).ti,ab,kf. | 155100 |
| 23 | (inappropriate* adj2 (prescrib* or prescription*)).ti,ab,kf. | 3789 |
| 24 | (over prescrib* or over prescription* or overprescrib* or overprescription*).ti,ab,kf. | 1930 |
| 25 | unsafe*.ti,ab,kf. | 11746 |
| 26 | ((clinician* or doctor* or pharmac* or physician* or practitioner* or prescriber* or provider*) adj3 (efficiency or performance or productivity)).ti,ab,kf. | 8247 |
| 27 | ((clinician* or doctor* or pharmac* or physician* or practitioner* or prescriber* or provider*) adj3 (accept* or adopt* or embed* or employ* or implement* or integrat* or promot* or take up or took up or uptake or usage or "use" or utili*)).ti,ab,kf. | 82471 |
| 28 | or/11-27 | 749095 |
| 29 | 10 and 28 | 1743 |

**Embase (Elsevier Embase.com)**

| 1 | 'electronic prescribing'/de | 4084 |
| --- | --- | --- |
| 2 | (eprescrib* OR eprescription*):ti,ab,kw | 1205 |
| 3 | ('e prescrib*' OR 'e prescription*'):ti,ab,kw | 1199 |
| 4 | (epharmacy OR epharmacies):ti,ab,kw | 64 |
| 5 | ('e pharmacy' OR 'e pharmacies'):ti,ab,kw | 88 |
| 6 | ((computeri?ed OR digital* OR electronic* OR internet OR online OR virtual*) NEAR/2 (prescrib* OR prescription*)):ti,ab,kw | 4708 |
| 7 | ((computeri?ed OR digital* OR electronic* OR internet OR online OR virtual*) NEAR/2 (pharmacy OR pharmacies)):ti,ab,kw | 1643 |
| 8 | (electronic* NEAR/2 (transmit* OR transmission OR send* OR sent) NEAR/2 (prescrib* OR prescription*)):ti,ab,kw | 93 |
| 9 | ((pharmac* OR prescrib* OR prescription*) NEAR/1 software*):ti,ab,kw | 726 |
| 10 | #1 OR #2 OR #3 OR #4 OR #5 OR #6 OR #7 OR #8 OR #9 | 9013 |
| 11 | 'medical error'/de | 20404 |
| 12 | 'medication error'/exp | 22936 |
| 13 | 'patient risk'/de | 10567 |
| 14 | 'patient safety'/de | 144041 |
| 15 | 'prescribing error'/exp | 8058 |
| 16 | 'safety'/de | 260547 |
| 17 | 'adverse event*':ti,ab,kw | 374037 |
| 18 | ((clinician* OR doctor* OR drug* OR medical OR medication* OR medicin* OR pharmac* OR physician* OR practitioner* OR prescrib* OR prescription* OR provider* OR treatment*) NEAR/3 (accident* OR error* OR inadvertent* OR mistake* OR uninten*)):ti,ab,kw | 37967 |
| 19 | (decei* OR fake OR fraud* OR misrepresent* OR scam* OR sham):ti,ab,kw | 154445 |
| 20 | ((drug* OR medical OR medication* OR medicin* OR patient* OR prescrib* OR prescription*) NEAR/3 (safe OR safeguard* OR safely OR safeties OR safety)):ti,ab,kw | 172025 |
| 21 | ((drug* OR medication* OR medicin* OR treatment*) NEAR/3 (misuse* OR overuse)):ti,ab,kw | 10034 |
| 22 | (harm OR harmed OR harmful* OR harms):ti,ab,kw | 199056 |
| 23 | (inappropriate* NEAR/2 (prescrib* OR prescription*)):ti,ab,kw | 6034 |
| 24 | ('over prescrib*' OR 'over prescription*' OR overprescrib* OR overprescription*):ti,ab,kw | 2908 |
| 25 | unsafe*:ti,ab,kw | 15516 |
| 26 | ((clinician* OR doctor* OR pharmac* OR physician* OR practitioner* OR prescriber* OR provider*) NEAR/3 (efficiency OR performance OR productivity)):ti,ab,kw | 11513 |
| 27 | ((clinician* OR doctor* OR pharmac* OR physician* OR practitioner* OR prescriber* OR provider*) NEAR/3 (accept* OR adopt* OR embed* OR employ* OR implement* OR integrat* OR promot* OR 'take up' OR 'took up' OR uptake OR usage OR 'use' OR utili*)):ti,ab,kw | 118834 |
| 28 | #11 OR #12 OR #13 OR #14 OR #15 OR #16 OR #17 OR #18 OR #19 OR #20 OR #21 OR #22 OR #23 OR #24 OR #25 OR #26 OR #27 | 1347648 |
| 29 | #10 AND #28 | 3600 |

**CINAHL with Full Text (EBSCOhost)**

| 1 | eprescrib* OR eprescription* | 78 |
| --- | --- | --- |
| 2 | "e-prescrib*" OR "e-prescription*" | 545 |
| 3 | epharmacy OR epharmacies | 2 |
| 4 | "e-pharmacy" OR "e-pharmacies" | 9 |
| 5 | ((computeri?ed OR digital* OR electronic* OR internet OR online OR virtual*) N2 (prescrib* OR prescription*)) | 1639 |
| 6 | ((computeri?ed OR digital* OR electronic* OR internet OR online OR virtual*) N2 (pharmacy OR pharmacies)) | 567 |
| 7 | (electronic* N2 (transmit* OR transmission OR send* OR sent) N2 (prescrib* OR prescription*)) | 50 |
| 8 | ((pharmac* OR prescrib* OR prescription*) N1 software*) | 168 |
| 9 | S1 OR S2 OR S3 OR S4 OR S5 OR S6 OR S7 OR S8 | 2701 |
| 10 | (MH "Medication Errors+") | 18152 |
| 11 | (MH "Patient Safety") | 73103 |
| 12 | (MH "Safety") | 30375 |
| 13 | (MH "Treatment Errors") | 9455 |
| 14 | "adverse event*" | 69958 |
| 15 | ((clinician* OR doctor* OR drug* OR medical OR medication* OR medicin* OR pharmac* OR physician* OR practitioner* OR prescrib* OR prescription* OR provider* OR treatment*) N3 (accident* OR error* OR inadvertent* OR mistake* OR uninten*)) | 31827 |
| 16 | decei* OR fake OR fraud* OR misrepresent* OR scam* OR sham | 28900 |
| 17 | ((drug* OR medical OR medication* OR medicin* OR patient* OR prescrib* OR prescription*) N3 (safe OR safeguard* OR safely OR safeties OR safety)) | 117104 |
| 18 | ((drug* OR medication* OR medicin* OR treatment*) N3 (misuse* OR overuse)) | 3777 |
| 19 | harm OR harmed OR harmful* OR harms | 60505 |
| 20 | (inappropriate* N2 (prescrib* OR prescription*)) | 4402 |
| 21 | "over-prescrib*" OR "over-prescription*" OR overprescrib* OR overprescription* | 1011 |
| 22 | unsafe* | 9149 |
| 23 | ((clinician* OR doctor* OR pharmac* OR physician* OR practitioner* OR prescriber* OR provider*) N3 (efficiency OR performance OR productivity)) | 4861 |
| 24 | ((clinician* OR doctor* OR pharmac* OR physician* OR practitioner* OR prescriber* OR provider*) N3 (accept* OR adopt* OR embed* OR employ* OR implement* OR integrat* OR promot* OR "take up" OR "took up" OR uptake OR usage OR utili*)) | 30651 |
| 25 | S10 OR S11 OR S12 OR S13 OR S14 OR S15 OR S16 OR S17 OR S18 OR S19 OR S20 OR S21 OR S22 OR S23 OR S24 | 352860 |
| 26 | S9 AND S25 | 1092 |

**Scopus (Elsevier Scopus.com)**

((TITLE-ABS-KEY("adverse event*" OR ((clinician* OR doctor* OR drug* OR medical OR medication* OR medicin* OR pharmac* OR physician* OR practitioner* OR prescrib* OR prescription* OR provider* OR treatment*) W/3 (accident* OR error* OR inadvertent* OR mistake* OR uninten*)) OR decei* OR fake OR fraud* OR misrepresent* OR scam* OR sham OR ((drug* OR medical OR medication* OR medicin* OR patient* OR prescrib* OR prescription*) W/3 (safe OR safeguard* OR safely OR safeties OR safety)) OR ((drug* OR medication* OR medicin* OR treatment*) W/3 (misuse* OR overuse)) OR harm OR harmed OR harmful* OR harms OR (inappropriate* W/2 (prescrib* OR prescription*)) OR "over-prescrib*" OR "over-prescription*" OR overprescrib* OR overprescription* OR unsafe* OR ((clinician* OR doctor* OR pharmac* OR physician* OR practitioner* OR prescriber* OR provider*) W/3 (efficiency OR performance OR productivity)) OR ((clinician* OR doctor* OR pharmac* OR physician* OR practitioner* OR prescriber* OR provider*) W/3 (accept* OR adopt* OR embed* OR employ* OR implement* OR integrat* OR promot* OR "take up" OR "took up" OR uptake OR usage OR utili*)))) AND (TITLE-ABS-KEY(eprescrib* OR eprescription* OR "e-prescrib*" OR "e-prescription*" OR epharmacy OR epharmacies OR "e-pharmacy" OR "e-pharmacies" OR ((computeri?ed OR digital* OR electronic* OR internet OR online OR virtual*) W/2 (prescrib* OR prescription* OR pharmacy OR pharmacies)) OR (electronic* W/2 (transmit* OR transmission OR send* OR sent) W/2 (prescrib* OR prescription*)) OR ((pharmac* OR prescrib* OR prescription*) W/1 software*)))) AND NOT ((INDEX(medline)) OR (INDEX(embase)))

3,077 results prior to MEDLINE and Embase overlap removal

1. results exported after MEDLINE and Embase overlap removal

**Supplementary Material File 2: Data Extraction Form**

**Metadata**

- PMID (if applicable)
- Open access URL (if available)
- Date of publication

**Study characteristics**

- Study design
- Location
- Prescriber setting
- Type of prescriber
- Is prescriber-patient interaction virtual or in person?
- Date range of data collection
- Population
- Sample size
- Intervention and comparison (if applicable)
- Use of theoretical framework (Y/N) - if Y, describe

**Primary outcomes**

- Description of e-prescribing software
- E-prescription software vendor
- E-prescription software owner/administrator and how it is housed
- Transmission mode
- Push vs. Pull model
- Outcomes of interest related to medication safety
- Outcome measure(s) - quantitative
- Outcome measure(s) - qualitative
- Results related to medication safety

**Secondary outcomes (if applicable; not present in every included study)**

- Secondary outcomes of interest related to efficiency/process improvement
- Secondary outcomes of interest related to uptake/implementation/adoption
- Outcome measure(s)
- Results related to secondary outcomes

**Supplementary Material File 3: Characteristics of included studies and e-prescribing systems**

| **Author, Year, Location** | **Study Design** | **Prescriber Setting, Type of Prescriber** | **E-prescribing Systems** | **Summary Statement** |
| --- | --- | --- | --- | --- |
| **QUANTITATIVE** | | | | |
| Farghali, 2021, Canada | Quantitative: secondary analysis/observational study with retrospective component | Not reported | - Not reported | Farghali et al (2021) conducted a secondary analysis of the 2016 National Survey of Community-based Pharmacists: Use of Digital Technology in Practice, that was collected via a web-based survey in collaboration with Canada Health Infoway and the Canadian Pharmacists Association. Community pharmacists from Ontario, Quebec, Saskatchewan, Alberta and British Columbia were the target population for the survey. Of the sample of 450 surveys, 66% of pharmacists reported e-prescribing to be a useful tool to reduce medication errors and 70% indicated that e-prescribing would improve efficiency in pharmacies. |
| Franklin 2014, UK, England | Quantitative: naturalistic stepped wedge study (Quasi-experimental) | Community pharmacies, pharmacists and dispensers | - Electronic Prescription Service (EPS) for transmitting prescriptions from primary care clinics to community pharmacies. - Push - Integration with EMR not reported | In a naturalistic stepped wedge study comparing electronic-to-paper and electronic-to-electronic (Electronic Prescription Service Release 2 [EPSR2]) prescriptions sent from primary care clinics to community pharmacies in the United Kingdom, Franklin et al. (2014) investigated the prevalence of content and labelling errors between types of prescriptions and fitted multivariable mixed effects logistic regression models for each outcome measure. Initially, they found a statistically significant 46% increase (CI 21% to 76%) in labelling errors for EPSR2 items compared to non-EPSR2. However, upon further investigation, they found that the source of 158 errors involved removing indications from labels, and 155 of these instances occurred at one pharmacy. After conducting a sensitivity analysis in which these 158 instances were not classified as errors, there was no longer a statistically significant effect of EPSR2 on labelling errors. |
| Kivekas 2016, Finland | Quantitative: survey based | Primary care, physicians | - Prescriptions in Finland transferred electronically to a centralized database called the Prescription Centre - Software vendor:  Effica (Tieto) and the Pegasos (CGI) - Pull - EHR integration | This study consisted of a web-based survey to assess general practitioners’ experience of an e-prescription system in Finland. In Finland, prescriptions are sent from the physician’s office to the ‘Prescription Centre’, a national centralized databased, and prescriptions can be dispensed in any pharmacy. A total of 69 surveys were completed and found that the majority (two-thirds) of general practitioners report e-prescribing to be safer than handwritten prescriptions. The participants reported some technical challenges related to software delays and browsing too many pages to write a prescription. |
| Kauppinen, 2017a, Finland | Quantitative: cross-sectional survey | Community pharmacies, pharmacists and dispensers | - Prescriptions in Finland transferred electronically to a centralized database called the Prescription Centre - Pull - Integration with EMR not reported | This cross-sectional postal survey was conducted in Finland in 2014 to explore pharmacists’ opinions regarding the impacts of electronic prescriptions (e-prescriptions) on medication safety in Finnish community pharmacies. A total of 778 questionnaires were completed and results showed that e-prescriptions in Finnish communities have improved medication safety, including lower number of prescription forgeries, decreased risk of dispending errors, improvement patient medication management, improved monitoring of duplicative therapy and drug interventions, and decreased risk of incorrect prescription interpretation. Despite these benefits, pharmacists encounter ambiguities and errors on a weekly basis. These can delay dispensing and sometimes cause serious risks to medication safety. |
| Ababneh 2020, Jordan | Quantitative: cross-sectional observational | Outpatient pharmacies,  Physicians | - E-prescription record contains all information need to fill, label and dispense a prescription - Push/pull not reported - EMR integration | In this cross-sectional observational study, Ababneh et al. (2020) in Jordan were interested to see if there are any differences in medication errors including prescription and dispensing errors when comparing a paper-based to an electronic prescribing system. What they found was that in the paper based system, out of the 2500 prescriptions, there were 3714 medication errors of which only 7.8% were prescription errors and the rest were dispensing errors. 4.4% of the errors were due to an inappropriate dose, concentration, quantity, route of administration and/or frequency. 1.6% of errors were due to drug interactions, 1.2% were inappropriate drug for that indication and 0.6% were duplicate drugs. In the electronic prescribing system, out of 2500 prescriptions, there were 631 medication errors and of those only 36.6% were prescription errors. 17.7% were drug interactions, 7.9% were duplicate drugs and 4.9% were inappropriate drugs for the specific indication. |
| Hitti 2017, Lebanon | Quantitative: pre and post intervention study (experimental) | Emergency Department, physicians | - Electronic discharge tool which includes e-prescribing capabilities - Push/pull not reported - EMR integration not reported | In a pre- / post-intervention study comparing emergency department prescription errors before and after the implementation of an electronic discharge tool with e-prescribing capabilities, Hitti et al. (2017) counted and classified errors in handwritten (HW) prescriptions vs. e-prescriptions. Errors were classified in two ways: whether they were due to incorrect information, missing information, or illegibility, and whether they were high-risk or low-risk. Overall, e-prescriptions were significantly associated with a reduced error rate (67.7% vs 45.5%, p<0.0001) (OR=0.40, 95%,CI [0.34–0.46]), particularly a reduction in missing information errors, illegibility and low-risk errors, and after adjusting for potentially confounding factors, e-prescriptions were a strong predictor of fewer errors (aOR = 0.40, 95%CI [0.35 – 0.47], p<0.0001).  However, e-prescriptions were associated with a borderline statistically significant increase in incorrect information errors (8.9% vs. 7.0% in HW prescriptions, p=0.05) and a statistically significant increase in high-risk errors (18.2% vs. 15.0% in HW prescriptions, p<0.0001). |
| Peikari 2015, Malaysia | Quantitative, cross-section self-report survey | Not stated, not stated | - Not reported | In Peikari et al., (2015), there was a positive significant relationship between e-prescribing ease of use and its positive impacts on pharmacists' outcomes (including facilitation of care, communication, workload, and error reduction), determined by examining the extent to which second generation e-prescribing usability improves the positive outcomes (including the improvement of communication, facilitation of providing care, reduction of medical errors and workload) amongst community pharmacists. |
| Khan 2020, Saudi Arabia | Quantitative: cross-sectional study | Outpatient clinics,  Physicians | - Not reported | In this cross-sectional study, Khan et al. (2020) tracked the medication errors that occurred with e-prescriptions for outpatients. Over a period of 12 weeks, out of the 329 prescriptions there were 29 prescriptions with incomplete patient information, 23 prescriptions with wrong or no dose, 6 prescriptions with wrong duration and 8 with missing durations, 23 prescriptions with dosing errors including missing dosing, and 52 drugs interactions of which only 3 were serious. Other types of errors included inappropriate selection of drugs, wrong/missing route of administration, duplication of therapy and lack of clear directions. |
| Hammar 2010, Sweden | Quantitative: survey | Physician’s office,  Physicians | - National Online Prescription Repository, a national mailbox for ePrescriptions - Pull - Not integrated with EMR | In this web-based survey study of Swedish pharmacists' attitudes of e-prescribing, Hammar (2010) surveyed 259 pharmacists and found the majority perceive e-prescribing to be safe for patients, improve communication between providers and patients, cost-effective, convenient and time-saving. Several system improvements were identified including improved coordination between the healthcare system and the pharmacies, and increased capabilities for providers to view and cancel e-prescriptions. |
| Rahimi, 2011, Sweden | Quantitative: cross-sectional design | Primary care,  Physicians | - Integrated electronic prescribing system (IEPS) collaboration with hospitals, primary healthcare centers (PHCs), and the Swedish National Pharmacy Corporation - Pull - Integration with EMR not reported | Rahimi et al. (2011) looked at pharmacists’ views on an integrated electronic prescribing systems with the link to pharmacological safety. There are more than 25 million e-prescriptions processed each year in Sweden are electronically transmitted from the physician's office to pharmacies. Respondents agreed that the risk for errors was reduced by using the system (mean score 3.83; 95% CI 3.53-4.11) Not only do pharmacists perceive the system be faster but pharmaceutical safety is also thought to be improved. |
| Weingart 2009, US, Boston | Quantitative: retrospective analysis | Ambulatory care,  Physicians | - PocketScript, an e-prescribing application developed by ZixCorp (Dallas, Texas) that includes patient medication profile and alerts prescriber of medication interactions - Push - EMR integration not reported | Weingart et al (2009) conducted an empirical model to estimate the potential impact of medication safety alerts on patient safety, health care utilization, and costs in ambulatory care. They found that electronic prescribing alerts may prevents serious adverse drug events and subsequent patient injury. Further, electronic prescribing alerts may reduce healthcare costs. |
| Kaushal 2010, US, New York | Quantitative: non-randomized clinical trial (quasi-experimental) | Adult primary care,  Physicians | - Commercially available e-prescribing system Providers had access to information in the EHR, drug alerts, patient insurance eligibility and formulary compliance were also checked by the system. - Push - EMR integration not reported | The purpose of this study was to assess the impact of e-prescribing system on the rates of ambulatory prescribing errors. In 7 primary care practices a stand-alone e-prescribing system was implemented. In the adopter group there was a rate of 35% of prescriptions that contained one error and in non-adopters there was a rate of 29.8% of prescriptions that contained one error. |
| Abramson 2011a, US, New York | Quantitative: Prospective, pre-post test (quasi-experimental) | Primary Care,  Physicians (MD and DO), Nurse Practitioners, Physician Assistant | - Commercially available e-prescribing system Providers had access to information in the EHR, drug alerts, patient insurance eligibility and formulary compliance were also checked by the system. - Push/pull not reported - EMR integration | Abramson et al. (2011a) conducted a nonrandomized pre-post study design, comparing 6 providers who used a commercial e-prescribing system and 15 providers who remained paper-based. They found that errors rates for e-prescribers decreased 1.5-fold and were significantly lower than non-e-prescribers at one year. All illegibility errors were eliminated by e-prescribing. |
| Abramson 2011b, US, New York | Quantitative: prospective case study | Academic-affiliated internal medicine ambulatory practice,  Physicians | - The Certification Committee for Health Information Technology certified commercial EHR with e-prescribing. Providers can create lists of frequently used orders and order sets - Push/pull not reported - EMR integration | Studying the transition between an older EMR system and a newer system, Abramson et al. (2011b) assessed the effect of the new system on the rates and types of prescribing errors. The baseline amount of errors pre-implementation of the new system was 1298, after 12 weeks of implementation 1331 and after 1 year was 1303. The largest change was in inappropriate abbreviation errors and non-abbreviation prescribing errors. A lesson learned from this study was that more providers need to be trained in EMR and e-prescribing to make this transition safer. |
| Moniz, 2011, US, Boston | Quantitative: experimental design | Community pharmacies, physician | - SureScripts uses the National Council for Prescription Drug Program standards within a secure electronic network; provides prescribers with dispensing histories and formulary and eligibility data - Push/pull not reported - EMR integration not reported | Moniz et al. (2011) conducted this controlled, before and after study was conducted in the US in 2006 and measured the effect of electronic prescribing on dispensing errors. The e-Prescribing system SureScript is a national pharmacy information exchange network that facilitates electronic prescribing and refilling of prescriptions. The study found E-prescribing from physician's office to a pharmacy decreased the risk of prescription and dispensing errors (i.e., Wrong dose, Wrong frequency, Wrong strength, Inappropriate "as directed" label, "as needed" missing or added, Wrong route) when compared to clinics generating a prescription electronically, printing it out, and giving it to the patient to take to the pharmacy. |
| Nanji 2011, US | Quantitative: retrospective cohort study | Primary Care, physicians | - Outpatient e-prescribing systems that are used in physician offices - Push/pull not reported - EMR integration not reported | In Nanji et al., (2011), outpatient computerized prescribing systems across three States were included to identify incidence of medical errors; potential adverse drug events; and rate of prescribing errors by error type and by prescribing system. In a sample of 3850 prescriptions, 452 (11.7%) contained a total of 466 errors. Researchers classified 163 (35.0%) of these errors as potential ADEs, so 4.2% of prescriptions contained potential ADEs. Of the potential ADEs, 95 (58.3%) were significant, 68 (41.7%) were serious, and none were life-threatening. The most common cause for error was omitted information (60.7% of total errors and 50.9% of potential ADEs), including duration, dose, or frequency. Omitted dose was the most likely to result in a potential ADE, accounting for 35% of all potential ADEs in the sample. |
| Bergeron 2013, US | Quantitative: interviewer-assisted survey | Primary care clinic,  Physicians | - Not reported | Bergeron et al. (2013) examined the differences for patients before and after implementation of e-prescribing. They conducted 3 waves of patient interviews; to e-prescribing (n=144) 6 months after e-prescribing (n=127) and 12 to 18 months after e-prescribing (n=73) with patients receiving new prescriptions. Descriptive statistics were calculated for all variables, with an exploratory analysis looking at patients understanding of instructions (indication/dosing) Understanding of indication was overall reduced (95.4% prior to e- prescribing, 97.9% 6 months after e-prescribing, and 89.8% 12 to 18 months after e-prescribing (p <0.05) as well as understanding of dosing (69.0% before e-prescribing, 67.1%  6 months after e-prescribing and 51.9% 12 to 18 months after e-prescribing (p<0.05). Not picking up prescriptions, related to patient uptake of e-prescribing was non-significant - 6.9% before e-prescribing, 10.6% 6 months after e-prescribing, and 2.5% 12 to 18 months after e-prescribing (P = . 07). Patient-reported reasons for not picking up prescriptions included medication cost/lack of insurance coverage (20.7%), choosing over-the-counter medication (17.3%), wanting to see if condition improved (24.2%), and side effect concerns (3.4%). |
| Cochran 2014, US | Quantitative: Retrospective cross-sectional study | Ambulatory care clinics, not stated | - Clinic e-prescribing software where pharmacy and family medicine clinic used integrated software - Push/pull not reported - EMR integration not reported | Cochran et al. (2014) studied discrepancy rates in e-prescription in 3 ambulatory clinic-community pharmacies. A total of 602 prescriptions written by 33 prescribers for 408 patients were evaluated from the 3 clinics. The discrepancy rate between the prescriber’s note and the e-prescription was 1.7%, 0.6% and 3.9% for the three clinics. The discrepancy rate between the e-prescription (clinic) and the prescription label (pharmacy) was 4.2%, 0.9% and 1.5%. Differences between directions for administration was the most common type of discrepancy identified. |
| Caruso 2015, US | Quantitative: Retrospective chart review | Emergency Department,  Mix: Emergency Department providers | - Software vendor: EmSTAT MD system Version 1.30 (Allscripts, Chicago, Ill) - Push/pull not reported - EMR integration | This study by Caruso et al. (2015) analyzed and classified reasons why retail pharmacies need to contact the pediatric emergency department for clarification on outpatient e-prescriptions. Through a retrospective chart audit, this study analyzed 49,583 e-prescriptions and found that they most common errors included administrative/insurance issues (47%; medication not covered by insurance, patient unable to afford medication) and prescription writing errors (41%; drug issues, incorrect dose for weight, unclear instructions).  Identifying the type of errors lead to discovery that 96% of prescriptions were unlikely to be filled by the pharmacy and required a call-back to the emergency department prescriber to clarify errors like concentration of drug, dose, medication product change due to lack of stock. |
| Bhavsar 2019, US | Quantitative: retrospective analysis | Not specified,  Physicians | - Not reported | In this study, Bhavsar et al. (2019) were interested to see if the incidence of hospitalization from adverse drug events has any relation to physician electronic prescribing in 9 US states. The counties that had the lowest amount of e-prescribing adoption had higher odds of adults 65 and older being hospitalized for a adverse drug event in comparison to a county with a high e-prescribing adoption rate (OR 1.10 95% CI 1.02-1.19). |
| Zheng 2021, US | Quantitative: retrospective, observational cohort analysis | Primary Care,  Physicians | - Not reported | Prescribers often use the free text feature when using e-prescribing, however the directions that are sent to the pharmacy aren't always clear. This leads to pharmacy staff often intervening and editing the text before it reaches the patient. In this retrospective observational cohort analysis, researchers Zheng et al. (2021) examined 529 990 e-prescriptions and examined how pharmacy staff edited the prescriptions and the quality before and after transcribing the directions in sample of 966 patient directions. Before the pharmacy staff edit the directions, 51.4% of sample directions had had at least one quality issue and after transcription only 11.3% had at least one quality issue. 79.5% of the prescriptions had their quality issues resolved by pharmacy staff. |
| Shah 2016, Not reported | Quantitative survey/cross-sectional | Hospitals, community physicians | - Not reported | Shah and Peikari (2016) used a system usability model to propose nine hypotheses, five of which were related to reducing prescribing errors. They used a cross-sectional self-report survey consisting mainly of Likert scale questions, answered by 188 GPs. Statistical testing included a bootstrapping test to show the relationship between reduction of prescribing errors and other variables. Results supported the following hypotheses: the usability of e-prescribing systems reduces prescribing errors (p< 0.05), the information quality generated by e-prescribing systems reduces prescribing errors (p< 0.01), consistency between e-prescribing systems’ user interfaces reduces prescribing errors (p< 0.01), and reduction of mental workload reduces prescribing errors (p< 0.001). Their hypothesis that error prevention in e-prescribing systems reduces prescribing error was not supported (p > 0.05). Ease of use of e-prescribing systems was significantly predictive of the reduction of users’ mental workload (p<0.01) and prescribing errors (p<0.05). |
| **QUALITATIVE** | | | | |
| Kauppinen 2017b, Finland | Qualitative: interview | Primary care, physicians | - Prescriptions in Finland are transferred electronically by a physician to a centralized database called the Prescription Centre - Pull - EMR integration not reported | This qualitative study by Kauppinen et al. (2017b) explored primary health care physicians’ experiences with the impacts of e-prescription on prescribing and medication safety in Finland. In Finland, e-prescriptions are stored in the ‘Prescription Centre’ a national centralized databased, and prescriptions can be dispensed in any pharmacy. Forty-two physicians participated in the qualitative interviews and identified some medication safety benefits to e-prescribing, including controlling medicines affecting the central nervous system. There are some challenges with the e-prescribing system including technical issues.  Additionally, e-prescribing has some problems related to efficiencies which impacts its ability to be used for management of a patient's overall medication. |
| Dyb 2019, Norway | Qualitative: interview | Not specified,  Physicians | - National e-prescribing system in Norway, provides information about all the prescribed pharmaceuticals that patients have received   from pharmacies.   - Not reported - Integrated with Summary Care Record | The Norwegian government has had a vested interest in improving medication safety via the implementation of e-prescribing practices. Dyb et al. (2019) conducted various qualitative interviews with professionals that work with the e-prescribing system or the Summary Care Record system to show how even with having access to a patient's medications is not enough to eliminate medication errors. The interviews highlighted that even though practitioners may have access to a shared health record, there can be medication errors when they don't check the patient's history. This is much more noticeable in e-prescriptions as opposed to paper ones. So while these tools (e-prescribing and a shared health record) have the potential to reduce medication errors, implementing changes in work practices is essential. |
| Josendal 2021, Norway | Qualitative - interview | Primary Care,  Physicians | - Prescriptions are sent to a central database called the Prescription Mediator (software vendor) - Pull - EMR integration not reported | In Norway, e-prescriptions have been adopted for some time, however the multi-dose dispensing (MDD) is still using a paper or fax system. Josendal et al. (2021) conducted a qualitative study in which they interviewed nurses and pharmacists to better understand the work and workflow around the medication handling process once e-prescribing was implemented. What they found was that there was more work for the nurses, as they had to double check the MDD, but this increased patient safety. In addition, e-prescribing allowed for less manual corrections of the MDD bags and faster changing of the MDD. The addition of electronic messaging between the GPs and pharmacists has allowed them to clarify prescriptions much more efficiently. Also, now with the implementation of e-prescribing MDD, pharmacists will be aware of medication changes that are even outside of the MDD, like antibiotics for example and can see prescriptions from both GPs and hospital doctors allowing them to help coordinate their care. This allows them to complete more prescription checks and overall improves patient safety. |
| Grossman 2007, US | Qualitative: interview | Primary Care,  Physicians | - E-prescribing module of an electronic health record, multiple software vendors - Push/pull not reported - EMR integration | This qualitative interview study by Grossman et al. (2007) examined physician experience of using e-prescribing software and found improved prescribing safety, quality, and efficiency through improved legibility, better documentation, reduced pharmacy call-backs, and improved management of renewals. |
| Bramble 2013, US, Nebraska | Qualitative: focus groups | Ambulatory care,  Mix: Family practice physicians, physician assistants, and advanced practice registered nurse | - Electronic health record with e-prescribing system - Push/pull not reported - EMR integration | This qualitative study conducted by Bramble et al. (2013) explored nurse and physician perspectives and perceptions about EHR with eRx and identified patient safety risks and benefits, which differed by clinician group. |
| Frail 2014, US | Qualitative: interview | Not stated, not stated | - Push - EMR integration | In this qualitative study, Frail et al. (2003) interviews 12 patients about their experience with e-prescription. Patients described positive and negative perceptions towards e-prescribing and felt adherence-related discussions and review of their current medications were not emphasized as a result of e-prescribing and there was a lost opportunity for interacting with community pharmacists about their medications during typical prescription drop-off. Patients could benefit from additional information such as having medication name and dosage before going to pharmacy and printed after-visit summaries. |
| Odukoya 2014, US | Qualitative: Observational and interview | community pharmacies, not stated | - 3 pharmacy information systems allowing integration of e-prescriptions received from prescriber offices into their computer system - software vendors:PDX, Pharmaserv, and Rx30 - Push/pull not reported - EMR integration not reported | Odukoya et al. (2014) directly observed and interviewed community pharmacists receiving e-prescriptions at five pharmacies in Southwest Wisconsin. During observation, pharmacists were asked to describe when and how they detected a prescription error and where it originated. In follow-up interviews, pharmacists were asked to recall past experiences with e-prescription errors. Over a period of 45 hours, researchers observed 74 e-prescription errors. Pharmacists described encountering e-prescription on a daily or weekly basis, and described 107 errors during interviews. The most common type of errors described were wrong drug quantity (40%), wrong duration of therapy (21%) wrong dosing directions (19%), and wrong dosage formulation (11%). Pharmacists attributed most errors to the prescribers, mainly due to auto-population of information and inadvertent entry or selection of wrong information, and perceived that prescribers therefore had more control over minimizing e-prescription errors. They also commented that mismatch of information could occur due to technological incompatibility between the prescribers' and pharmacies' computer systems. |
| Yang 2018, US | Qualitative: retrospective qualitative analysis | Ambulatory care,  Physicians | - 501 different e-prescribing/electronic health record software applications across U.S. - Push/pull not reported - Some were integrated with EHR systems | Yang et al. (2018) assessed the variability and quality of free-text prescriber instructions (Sig) within e-prescribing and electronic health record software. They completed a retrospective qualitative analysis on a sample of 25,000 e-prescriptions issued by 22,152 community-based prescribers using 501 different electronic health record or e-prescribing software. The research team sorted and classified the Sig data. Variation in Sig data was noted, much which could be avoided. 10.1% contained at least 1 quality related event that would likely produce confusion and waste time at pharmacies related to time spent clarifying - calculated kappa coefficient of κ=0.72. This confusion could lead to patient safety issues. |
| **MIXED METHODS** | | | | |
| Sweidan 2010, Australia | Mixed-methods: key informants interviews + modified Delphi | General practice and public health, not stated | - Not reported | Sweidan et al. (2010) conducted a multi-methods study. An expert panel rated a list of 114 e-prescribing features (adapted Delphi methods) and selected 27 features as having high positive impact on patient safety, quality of care, usefulness to clinician, usefulness to the patient. The high rated features should be considered for integration into e-prescribing software to support safety and quality. |
| Elliott 2016, Australia | Mixed-methods: retrospective audit, focus groups, interviews | Residential aged care facility,  Physicians | - e-prescribing and medication management system (ePMMS) - Push - EMR integration not reported | Elliott et al (2016) explored the perspectives of nurses, GPs, and pharmacists regarding e-prescribing and medication management system (ePMMS). They held focus groups with GPs (n=5) and nurses (n=12) and interviews with pharmacists (n=2). Thematic analysis was completed on the qualitative data, finding safety benefits identified by the members of the healthcare team. Nurses felt that the there was a decrease in errors/delays with ordering/discontinuing medications due to the ePMMS, pharmacists felt safety was improved as they didn’t have to interpret doctors handwriting and GPs reported safety benefits where they can make changes from the clinic that are in sync with the facility. Additionally, increased efficiency in some cases (when prescriber away from facility) with limitations and barriers including poor uptake, thought to be related to (GPs) having fewer patients at the facility, inefficiency related to it being faster to handwrite while at facility, ePMMS, infrequent use of mobile devices/dislike of mobile screen, different e-prescribing software at different facilities, and need for paper copies (signature/medication coverage purposes). |
| VanLaere 2022, Belgium | Mixed-methods: retrospective analysis and interview | Unknown,  Physicians | - Recip-e service of the national eHealth platform. For traceability /feedback mechanism, a paper proof of the e-prescription is still given to patient - Push - EMR integration not reported | Belgium has had e-prescribing for the past 6 years. VanLaere et al. (2022) were interested in finding the discrepancies between the e-prescriptions and actual dispensing and conducted a mixed-methods study. They consulted stakeholders to identify warning flags which were then quantified in a random set of e-prescriptions. Lastly, they conducted qualitative interviews with stakeholder to explain the errors. They identified 15 warning flags and after examining 11 798 e-prescriptions the most common errors were related to the construction of the prescription (18.88%), dispensing extra products (3.81%) and dispensing product that didn't exactly match the product prescribed (3.39%). From the interviews, the cause of these issues can be attributed to the software the prescriber is using (i.e. using free text as opposed to the structured prescription functionality) and differences in medication databases that prescribers and pharmacists use. |
| Lapane 2011, US, Florida, Massachusetts, New Jersey, Nevada, Rhode Island, and Tennessee | Mixed methods: surveys, focus groups | Ambulatory care,  Physicians | - SureScripts LLC (the nation's largest e-prescribing network), Multiple software vendors - Push - EMR integration not reported | In a mixed methods study Lapane et al. looked at patient safety within e-prescribing. 157 clinicians completed a web-based survey and 276 clinicians participated in 64 focus groups. The majority of providers (78%) felt that e-prescribing was much better than other methods for patient safety and quality of care. The qualitative findings highlight several patient safety advantages (I.e., medication reconciliation, reduced errors due to illegibility of handwriting) and patient safety concerns (validity and completeness of medication information, technology concerns). |
| Hincapie 2019, US | Mixed methods: retrospective analysis | Not specified,  Physicians | - Not reported | Hincapie et al. (2019) completed a retrospective quantitative and qualitative analysis of voluntarily reported e-prescribing related incidents. The study used a convenience sample from two incident reporting software, with 589 incident reports from the PEER Portal, and 550 incident reports from the PQC. Descriptive statistics were calculated for all variables of interest. A random sample of comments in the text field were analyzed qualitatively using a conceptual framework. The most frequent types of e-prescribing problems found in the PEER Portal data and PQC data were in patient directions (21.9%/23.3%), incorrect drug (10%/ 14.7%), incorrect dose (9.3%/ 11.6%, and incorrect quantity (16.5%/6.0%). Thematic analysis highlighted the increased risk to patients if they are prescribed the wrong therapy. E-prescribing incidents may lead to interruptions in the pharmacy workflow, creating inefficiency and extra work due necessary clarifications with prescribers. |

**Supplementary Material File 4: Medication Safety Outcomes**

| **Author/ Year/ Location** | **Outcome of Interest** | **Outcome Measure**   **(QUAL/ QUAN/ MMR)** | **Findings (+,-)** |
| --- | --- | --- | --- |
| Ababneh, 2020,    Jordan | Prescription Error  Dispensing Error | QUAN: Frequency Count | **+** 631 medication errors in e-prescriptions compared to 3714 in paper prescriptions. Reduction in prescription error from 288 in paper prescriptions to 231 in e-prescriptions due to inappropriate drug for indication, duplication of drugs on same prescription, or prescription of drugs that could have a drug-drug interaction. Reduction in dispensing error from 3426 in paper prescriptions to 400 in e-prescriptions due to drug omission, inappropriate or inadequate labelling, or dispensing the wrong drug, dose, or dosage form. |
| Abramson, 2011a,    US (a) | Prescription Error | QUAN: Frequency Count | **+** Error rates for e-prescribing adopters decreased 1.5-fold, from 26 errors per 100 prescriptions at baseline to 16 errors per 100 prescriptions at one year (p= 0.09). Error rates for e-prescribing adopters were significantly lower than for non-adopters at one year    (p < 0.001). Illegibility errors were high at baseline and eliminated by e-prescribing. |
| Abramson, 2011b,    US | Prescription Error  Adverse Drug Events | QUAN: Frequency Count | **+** Error rates were highest at baseline (35.7 per 100 prescriptions) and significantly lower at 12 weeks (21.1 per 100 prescriptions, p < 0.001) and one year (12.2 per 100 prescriptions, p < 0.001) post-implementation. Inappropriate abbreviation errors were also highest at baseline (24.1 per 100 prescriptions) and significantly lower at 12 weeks (10.6 per 100 prescriptions, p < 0.001) and 1 year (5.9 per 100 prescriptions, p <0.001) post-implementation.  **-** Rates of near misses and rule violations did not significantly differ between time periods. No preventable adverse drug events were detected. At all three time periods, inappropriate abbreviations constituted most of the prescribing errors. |
| Bergeron, 2013,    US | Patient Safety | QUAL: Interviews | Following implementation of e-prescribing patients experienced reduced understanding of medication indication/dosing (p =0.03) (95.4% prior to e-prescribing, 97.9% 6 months after e-prescribing, 89.8% 12 to 18 months after e-prescribing). Patient understanding of medication dosing was reduced (p=0.02) (69.0% before e-prescribing, 67.1% % 6 months after e-prescribing, 51.9% 12 to 18 months after e-prescribing). |
| Bhavsar, 2019,    US | Adverse Drug Event | QUAN: Frequency Count | **+** Counties with the lowest amount of e-prescribing adoption had greater odds of adults 65 and older being hospitalized for an adverse drug event in comparison to counties with high e-prescribing adoption rates (0.25 versus 0.22) (OR 1.10 95% CI 1.02-1.19). A total of 6400 out of 2,725,378 (0.23%) discharges were due to adverse drug events that occurred in the community. |
| Bramble, 2013,    US | Patient Safety  Prescription Error | QUAL: Focus Groups | **-** From perspective of nurses and providers electronic health records with e-prescribing adoption has led to patient safety concerns. 5 most common safety concerns include: 1. clinical alert fatigue, 2. inaccurate information being propagated over time, 3. reducing illegible prescriptions, 4. lack of generic drug names, and 5. dose error from drop down menus. |
| Caruso, 2015,    US | Administration Error  Prescription Error | QUAN: Frequency Count | Severe or serious call backs were the result of lack of automated dosage checks by the e-prescribing system. Drug dosage recommendations were not available through the e-prescribing system and medications written for milliliters instead of milligrams resulted in overdoses of 5 to 1000 times the normal dose. |
| Cochran, 2014,    US | Prescription Error | QUAN: Frequency Count | 602 prescriptions (written by 33 prescribers for 408 patients) had a discrepancy rate between the prescriber’s note and the e-prescription of 1.7%, 0.6% and 3.9% across three different clinics. Discrepancy rate between e-prescription (clinic) and prescription label (pharmacy) was 4.2%, 0.9%, and 1.5%. Differences between directions for administration was the most common type of discrepancy identified. |
| Dyb, 2019,    Norway | Medication Safety Error | QUAL: Interviews | Challenges of increasing implementation of e-prescribing and reducing medication errors includes flawed work practices such as failing to look up patients’ pharmaceutical history and issuing double prescriptions. |
| Elliott, 2016,    Australia | Prescription Error | QUAL: Focus Groups | **+** Nurses perceived use of e-prescriptions decreased errors/delays with ordering/discontinuing medications. Pharmacists perceived the use of e-prescriptions improved safety as they didn't have to interpret doctors handwriting. GPs reported safety benefits where they can make changes from the clinic that are in sync with the facility. |
| Farghali, 2021,    Canada | Medication Safety Error | QUAN: Survey | **+** Most pharmacists (66%) reported e-prescribing as a useful tool to reduce medication errors and 70% indicated e-prescribing would improve efficiency in pharmacies. |
| Frail, 2014,    US | Patient Safety | QUAL: Interviews | **+** Perceived reduction in medication errors (physician writing was illegible resulting in dosage error), increased access to information for prescribers, availability of after-visit summary. |
| Franklin, 2014,    UK | Prescription Error | QUAN: Frequency Count | **-** E-prescribing system did not reduce labelling errors compared to a paper-based system. |
| Grossman, 2007,    US | Patient Safety | QUAL: Interviews | **+** Improved features of e-prescriptions include improved legibility, clearly documented medication lists, integrated into electronic medical records for access to lab results and patient health information, and drug-drug safety alerts. |
| Hammar, 2010, Sweden | Patient Safety | QUAN: Survey | **+** 95% of respondents (246/259) report e-prescribing to be safe for patients, 93% report electronic storing of prescriptions to be safe for patients, and 96% report e-prescribing provides patient benefits. |
| Hincapie, 2019,    US | Prescription Error  Patient Safety | MMR:  QUAN: Frequency Count  QUAL: Thematic Analysis | Frequency and type of e-prescribing problems most found in two e-prescribing software include patient directions (21.9%/23.3%), incorrect drug (10%/ 14.7%), incorrect dose (9.3%/ 11.6%), and incorrect quantity (16.5%/6.0%). Thematic analysis of comments in the open text field found increased risk to patients if they are prescribed the wrong therapy. |
| Hitti, 2017,    Lebanon | Prescription Error  Patient Safety | QUAN: Frequency Count | **+** E-prescriptions significantly associated with a reduced error rate. |
| Josendal, 2021,    Norway | Patient Safety | QUAL: Interviews | **+** Perceptions of increased patient safety include: 1. prescribing was not well integrated within the system and lead to a lot of double-checking by home care nurses, 2. pharmacists being notified of changes to treatment, leading to more prescription checks, 3. shift from paper to electronic decreased the risk of patients seeking/ taking double doses of “addictive medications.” |
| Kauppinen, 2017a, Finland | Medication Safety Error  Prescription Error | QUAN: Survey | **+** Medication safety was improved by lower number of prescription forgeries, reduced risk of dispensing errors, better medication management. Many respondents (32%) reported weekly ambiguities or errors in e-prescriptions that required clarification during the dispensing process. Three most common ambiguities or errors included: 1. incorrect total amount of medication; 2. missing notification of exceptional dosage instructions or exceptional purpose of use; and 3. unclear or incorrect dosage instructions. |
| Kauppinen, 2017b, Finland | Medication Safety Error  Prescription Error | QUAL: Interviews | **+** Primary health care providers reported positive safety benefits to e-prescribing including convenience of e-prescriptions, easier prescribing and controlling narcotics and medicines, more readily available information about patients' prescriptions through the Prescription Centre.  **-** Technical problems in the e-prescribing system were described as hindering prescribing and incoherent prescription information in the Prescription Centre. |
| Kaushal, 2010,    US | Prescription Error  Adverse Drug Events | QUAN: Frequency Count | **+** E-prescribing significantly decreased prescription error rates from 42.5 to 6.6 per 100 prescriptions (p<0.001).E-prescribing adopters had significantly lower rates of errors and near misses than non-adopters at one year (6.6 vs. 38.4 errors per 100 prescriptions (p<0.001) and 1.3 vs. 2.7 near misses per 100 prescriptions (p<0.001)).  - There were 0.04 preventable adverse drug events per 100 prescriptions at follow-up, compared with zero at baseline (p = >0.99). Rates of preventable adverse drug events trended lower among adopters (0.04 vs. 0.26 per 100 prescriptions (p=0.26)). |
| Khan, 2020,    Saudi Arabia | Medication Safety Error  Prescription Error | QUAN: Frequency Count | Of 329 e-prescriptions, 29 prescriptions had incomplete patient information; 23 prescriptions had the wrong dose or no dose information; 6 prescriptions were the wrong duration; 8 prescriptions were missing duration information. Among 52 drug-drug interaction errors, 15 were minor, 15 were moderate, and 3 were serious. |
| Kivekas, 2016,    Finland | Patient Safety | QUAN: Survey | **+** Two-thirds of GPs regarding e-prescribing to be safer than using handwritten prescriptions for medications. |
| Lapane, 2011,    US | Patient Safety | MMR:  QUAN: Survey  QUAL: Focus Groups | **+** 78% of clinicians surveyed felt e-prescribing was much or somewhat better than other methods in terms of patient safety. Focus groups identified safety advantages including value in doing medication reconciliation at point of prescribing, knowing medications prescribed by other providers, remote access to patients’ medication lists, ability to share list of medications with patients, reduces errors due to illegibility of handwriting.  **-** Patient safety concerns included validity and completeness of medication information, accidentally prescribing duplicate medications, drop down menu issues resulting in wrong drug or dose being prescribed, small screen size on handheld devices leads to wrong drug or dose being prescribed, order of drop-down menus leads to wrong drug or dose being prescribed. |
| Moniz, 2011,    US | Prescription Error  Dispensing Error | QUAN: Frequency Count | **+** Electronic transmission of prescription data from physicians’ offices to a pharmacy nearly halved the risk of dispensing errors compared with generating the prescription with outpatient computerized physician order entry or using printed prescriptions. |
| Nanji, 2011,    US | Medication Safety Error  Adverse Drug Events  Prescription Error | QUAN: Frequency Count | Of 3850 prescriptions, 452 (11.7%) contained a total of 466 errors. Researchers classified 163 errors as potential adverse drug events with 95 (58.3%) considered significant, 68 (41.7%) serious, and 0 life-threatening. E-prescription error rates ranged from 5.1% to 37.5%. The most common cause for error was omitted information such as duration, dose, or frequency. Omitted dose was most likely to result in adverse drug event, accounting for 35% of all adverse drug events in the sample. |
| Odukoya, 2014,    US | Medication Safety Error | MMR    QUAN: Observation  QUAL: Interviews | During 45-hours of observation, 75 e-prescription errors were documented, and 107 e-prescription errors were described during interviews. All participants reported they encountered e-prescription errors daily (approximately 5 out of 100 e-prescriptions) or weekly (5 to 15 errors) in their practice setting. The 4 most common errors observed included: 1. Wrong drug quantity (40%), 2. Wrong duration of therapy (21%), 3. Wrong dosing directions (19%), and 4. Wrong dosage formulation (11%). |
| Peikari, 2015,    Malaysia | Medication Safety Error | QUAN: Survey | **+** Significant relationship between e-prescribing ease of use and pharmacists’ outcomes including facilitation of care, communication, workload, and error reduction. |
| Rahimi, 2011,    Sweden | Medication Safety Error | QUAN: Survey | **+** The respondents generally agreed that the risk for prescription errors was reduced by using the e-prescription system (mean score 3.83; 95% CI 3.53–4.11). Pharmacists agreed the e-prescription software was useful for generally improving patient safety (mean score 3.67; 95% CI 3.44–3.90) (Fig. 4). The index score for e-prescribing usefulness and usability for the pharmacological safety area was 3.74 (± 0.89), which was significantly higher (95% CI 0.49–0.99, p < 0.05) than the test value (score 3). |
| Shah, 2016,    Not Reported | Prescription Error | QUAN: Survey | **+** The usability of e-prescribing systems reduces prescription errors (p< 0.05), quality of information generated (p< 0.01), consistency between e-prescribing systems’ user interface (p< 0.01), and reduction of mental workload (p< 0.001). The hypothesis that error prevention in e-prescribing systems reduces prescribing error was not supported (p > 0.05). |
| Sweidan, 2010, Australia | Patient Safety | QUAN: Modified Delphi | **+** Positive features of e-prescribing systems include: 1. allow user to record patient clinical details, medication information, allergies and pregnancy and breastfeeding status; 2. record in a format used for decision support and displayed to the user; 3. record changes to discontinuation or changes of medication; 4. import and export clinical data from external sources; 5. provide user information on recommended therapeutic options and medication selection process; 6. display medication lists to differentiate between similarly named products; 7. provide information on medication dose and form and generic name; 8. alerts prioritized by clinical importance with warnings of patient allergy, interactions with other drugs. |
| VanLaere, 2022, Belgium | Prescription Error | MMR:  QUAN: Frequency Count  QUAL: Interviews | 15 different types of prescription errors were identified and categorized into 4 groups: 1. filings of medication, 2. metadata of the e-prescription and/or medications, 3. posology of information, 4. technical soundness. The most common prescription errors were related to the construction of the prescription (18.88%), dispensing extra products (3.81%), and dispensing product that did not match the product prescribed (3.39%). |
| Weingart, 2009,    US | Patient Safety | QUAN: Frequency Count  Modified Delphi | **+** E-prescribing was found to prevent serious adverse drug events and subsequent patient injury. Electronic drug alerts likely prevented 402 adverse events in 2006, including 49 potentially serious, 125 significant, and 228 minor events. Accepted alerts may have prevented a death in 3 cases, permanent disability in 14, and temporary disability in 39 cases. Alerts potentially resulted in 39 fewer hospitalizations, 34 fewer emergency department visits, and 267 fewer office visits, for a cost savings of $402 619 (USD). |
| Yang, 2018,    US | Prescription Error | QUAL: Thematic Analysis | Retrospective analysis on a sample of 25,000 e-prescriptions found 10% of e-prescriptions contained at least 1 quality related event that would likely produce confusion or inefficiency at pharmacies related to time spent clarifying (κ=0.72). The most common quality related event included dose and route. |
| Zheng, 2021,    US | Prescription Error | QUAN: Frequency Count | Before transcription, 497 (51.4%) e-prescriptions contained at least one quality issue. After transcription, 109 (11.3%) e-prescriptions contained at least one quality issue. This left 395 (79.5%) e-prescriptions with quality issues that were fully resolved by pharmacy staff transcription. |

**Supplementary Material File 5: Efficiency and process improvement outcomes**

| **Reference** | **Outcomes Related to Efficiency/Process** | **Outcomes Related to Uptake** | **Findings** |
| --- | --- | --- | --- |
| Kauppinen, 2017b, Finland | Interviews identified **system improvements** needed and **process inflexible**    **as well as technological impairments** | N/A | E-prescribing requires system improvements relating to prescription information (incoherent and not up to date), as well as process (inflexible and could be impacted by slow internet connection between electronic patient records and E-prescribing system). |
| Kivekas, 2016,    Finland | Surveys identified **perceived usefulness** and **perceived ease of use** | Surveys identified suggestions for **improving E-prescribing** | Findings indicated that E-prescribing is useful and easy to use. However, there are challenges with technology aspects. |
| Peikari, 2015,    Malaysia | Surveys identified **ease of use** and **increased communication** | Surveys identified **decreased workload** | Findings indicated that ease of use of E-prescribing has a positive influence on outcomes. Users can communicate well with each other using a capable system producing quality information. Higher quality information lowers pharmacist workload. |
| Caruso, 2015,    US | Frequency counts identified **inefficiency** | N/A | Findings reported that errors led to discovery that 96% of prescriptions unlikely to be filled by pharmacy, requiring clarification through call-back to prescriber. Prescription error follow up time for pharmacy and prescriber approximately 127 hours, with an approximate annual cost to prescriber department (ED) |
| Bramble, 2013, US | Focus groups identified **efficiency** and **workarounds** and **inefficiency** | N/A | Found that provider-reported inefficiency using EHR with E-prescribing system slower than handwritten due to system redundancies. Estimated reduced patient clinic visits could be possible using EHR with E-prescribing system.  Nurse-reported clinic efficiencies were found and workarounds were generated for patient notes. |
| Grossman, 2007, US | Interviews identified **efficiency** | N/A | Found that preparing E-prescriptions took about the same time as handwritten and eliminated staff time to help administer prescription in clinic. |
| Hincapie, 2019, US | Reports from error-reporting systems identified **inefficiency** | N/A | Findings reported e-prescribing incidents may lead to pharmacy workflow interruptions and cause inefficiency and more work due needed clarifications with prescribers. |
| Yang, 2018, US | Qualitative analysis identified **inefficienc**y | N/A | Found that E-prescriptions contained at least one quality related event that could cause inefficiency at pharmacies with time spent on clarifications. |
| Shah 2016 | Surveys identified e**ase of use** | N/A | Findings reported ease of use of E-prescribing system helped reduce workload and prescribing errors for users. |
| Elliott, 2016, Australia | Interviews and focus groups identified **efficiency shah** | Interviews and focus groups identified **uptake** | Found that general practitioners (GPs) felt E-prescribing system increased efficiency entering prescriptions away from facility. Nurses and pharmacists felt system saved time. However, GPs felt E-prescribing slower at facility compared to handwritten. Poor uptake for some GPs with few patients at facility, limited use of E-prescribing system and mobile system, and different software at facilities. |
| Bergeron, 2013, US | N/A | Descriptive statistics and exploratory measures (interviews and follow-up phone calls) identified prescription **uptake** | Not picking up prescriptions, related to patient uptake of e-prescribing was non-significant - 6.9% before e-prescribing, 10.6% 6 months after e-prescribing, and 2.5% 12 to 18 months after e-prescribing (P = . 07). Patient-reported reasons for not picking up prescriptions included medication cost/lack of insurance coverage (20.7%), choosing over-the-counter medication (17.3%), wanting to see if condition improved (24.2%), and side effect concerns (3.4%). |
| Rahimi, 2011, Sweden | Surveys indicated **work efficacy** and **usefulness** | N/A | Findings indicated pharmacists perceived E-prescribing system to be useful and user-friendly enough to support improved work efficacy. |
| Farghali, 2021, Canada | Surveys indicated **productivity** | N/A | Findings showed most pharmacist respondents indicated E-prescribing system would have positive impact on productivity in pharmacy. |
| Hammar, 2010, Sweden | Surveys indicated **efficiency** with time-saving**; system improvements positive;** better **communication** and relationships; **cost effective** | N/A | Findings showed that over half of pharmacist respondents reported E-prescribing to be time-saving and convenient, and contributed to better patient and prescriber relationship and communication. System improvements relating to increased healthcare facility and pharmacy coordination and better prescribing viewing and editing of E-Prescriptions were reported. Most respondents reported E-prescribing cost effective. |
| Weingart, 2009, US | Frequency counts and Modified Delphi approach indicated **healthcare utilization** |  | Found that E-prescribing alerts potentially resulted in fewer hospitalizations, ED visits and office visits and created cost savings. |
